# Traditional medicine usage among adult women in Ibadan, Nigeria: a cross-sectional study

**DOI:** 10.1186/s12906-020-02881-z

**Published:** 2020-03-20

**Authors:** Suellen Li, Stella Odedina, Imaria Agwai, Oladosu Ojengbede, Dezheng Huo, Olufunmilayo I. Olopade

**Affiliations:** 1 Department of Internal Medicine, Massachusetts General Hospital, Boston, MA USA; 2 Center for Population and Reproductive Health, College of Medicine, University of Ibadan, UCH, Ibadan, Nigeria; 3 Department of Public Health Sciences, University of Chicago, Chicago, IL USA; 4 Center for Clinical Cancer Genetics and Global Health, Department of Medicine, University of Chicago, Chicago, IL USA

**Keywords:** Traditional medicine, Herbal medicine, Global Health, Women’s health, Ethnic minorities

## Abstract

**Background:**

Previous research has revealed high rates of traditional medicine usage in Nigeria. Reports of widespread contamination of herbal medicine products and higher rates of noncompliance with Western medications among traditional medicine users have raised concerns about the safety of traditional medicine use. Few studies have explored how demographic factors predict rates of traditional medicine use in the general population.

**Methods:**

We conducted interviews of 748 adult women recruited from the communities in the city of Ibadan, Nigeria from 2013 to 2015. A structured questionnaire was created to collect data on rates of traditional medicine use and demographic factors such as age, education, ethnicity, and occupation. Multivariate logistic regressions were run to examine factors related to traditional medicine use, and the effects were measured with odds ratios (OR) along with 95% confidence interval (95%CI).

**Results:**

The overall proportion of traditional medicine use was 81.6%. Women from the Ibo and Hausa ethnic groups were significantly less likely to use traditional medicine than the majority Yoruba group (OR 0.25, 95%CI 0.10–0.63;, OR 0.43, 95%CI 0.24–0.76) respectively). In addition, educated women were less likely than their non-educated counterparts to have used traditional medicine, with the biggest effect seen in women with a secondary education (OR 0.42, 95%CI 0.21–0.85).

**Conclusions:**

We found a high rate of traditional medicine usage, consistent with that found in prior research. A novel finding was the significance of ethnicity as a predictor for usage rates.

## Background

The World Health Organization (WHO) estimates that 80% of the world population relies on complementary and alternative medicines (CAM), or traditional medicine, which includes all healing practices indigenous to different cultures. Traditional medicine is often juxtaposed with modern biomedicine. In Nigeria and other parts of West Africa, herbal remedies and spiritual healing are particularly common [1]. Reported uses for traditional medicine (TM) include cancer [2], diabetes [3], HIV [4], and hypertension [5], among others. Parents often treat their children with traditional medicine, with documented uses for epilepsy, asthma, and sickle cell disease [6]. These practices are not confined to their country of origin, however, and immigrants from Nigeria often bring their traditional beliefs to their new country of residence, where physicians are unfamiliar with their practices [7]. This is especially important in oncology, as multiple forms of CAM have been shown to interfere with chemotherapy regimens [8, 9].

Growing awareness of the prevalence of traditional medicine use has spurred biomedical efforts to test its effectiveness. Promisingly, a number of Nigerian herbal medicines have been tested and have demonstrated efficacy against malaria [10]. However, traditional medicine usage has also been shown to interfere with proper adherence to biomedical treatment regimes. Previous studies have found relationships between traditional medicine usage and medication non-adherence [4], and many patients turn to biomedical treatment only after traditional medicine has failed to cure them [11]. Unfortunately, a majority of patients do not disclose their use of traditional medicine to biomedical doctors, in large part because doctors fail to ask [2, 12]. A recent study of American oncologists found that only 26% initiate discussions about herbs and supplements with their patients, and that they significantly underestimate the percentage of their patients who use CAM. In reality, there is high CAM utilization among cancer patients ranging from 43 to 88%, and these numbers may be even higher in lower-income countries [13–15].

Despite the popularity of traditional medicine in Nigeria, there are few regulations on its use or quality control. One study of herbal medicines in Nigeria found that 100% of herbal medicine samples contained elevated amounts of heavy metals such as cadmium, lead, and mercury [16]. Other research has revealed levels of microbial contamination of herbal medicines that are unacceptably high by WHO standards [17]. This is concerning, as herbal medicine usage is widespread, and widely regarded as safe by its users [1].

Usage of traditional medicine is often attributed to its relative ease of access and affordability when compared to biomedicine. This is particularly relevant in the African continent, where the ratio of traditional healers to population can be 100 times greater than that of medical doctors to population [18]. A previous study in Ibadan, Nigeria found that people were more likely to choose traditional medicine because of perceived higher effectiveness, accessibility, and affordability [19]. Another cross-sectional study identified increased utilization of TM, and corresponding decreased utilization of biomedicine, in populations who believe in supernatural causes of disease [20].

While many studies have been performed on the use of traditional medicines in patients with a variety of health conditions, few have investigated the use of traditional medicine by healthy Nigerian adults [1]. Previous studies have identified influence of family or friends as an important determinant of traditional medicine use [1, 6]. However, there have been conflicting findings as to the importance of age, socioeconomic status, and education level in predicting an individual’s usage of traditional medicine [4, 5, 21]. Given both the prevalence of traditional medicine use among Nigerian populations, and potential danger of consuming contaminated herbal medicines, it is important to understand patterns of usage. This study aims to identify the prevalence of, and determinants of TM use among healthy adult women in the urban city of Ibadan, Nigeria.

## Methods

### Subjects

Seven hundred fourty-eight adult women were recruited using community-based methods from Ibadan, Nigeria from 2013 to 2015 as healthy controls for the Nigerian Breast Cancer Study (NBCS), a case-control study of breast cancer. These controls were recruited to match breast cancer cases recruited in the surgery and oncology departments according to Nigerian ethnicity and age group. The subjects were all adult females who were 18 years or older, absent of breast cancer, and able to give informed consent. The Yoruba participants were mainly recruited from the Akinyele LGA of Oyo State (Oboda, Sawmill, Abiola Quarters, Tybato, Aregbe, Idi-aba and Idi-Ose), where recruitment was done by house visits. The Hausa participants were recruited from communities in Sabo, and women interested in participating came to the Sabo Palace and were interviewed with the help of an interpreter. The Ibo and other ethnic group participants were recruited from Aleshiloye market, a major market in Ibadan. The recruitment methods and study design have been documented in detail elsewhere [22, 23]. All subjects were informed about the research and gave consent. This project was approved by institutional review boards at the University of Chicago and the University of Ibadan.

### Questionnaire

Interviews were conducted in person as part of epidemiological and demographic data collection for NBCS. Participants were informed that the questionnaire would ask about a variety of demographic and health-related factors. Interviews were conducted in either English or Yoruba by trained researchers. The questionnaire collected demographic data such as age, education level, ethnicity, and socioeconomic status, as well as health-related data such as body mass index (BMI), which was categorized into underweight (< 18.5), normal (18.5–24.9), overweight (25.0–29.9), and obese (30+) according to the World Health Organization categorizations [24]. Women were asked how their weight compared to their weight 1 year prior. Women were also asked whether or not they had used traditional medicine, the frequency of their usage, and their reasons for using it. Respondents could choose from several multiple-choice options to provide their reasons, and were also allowed to specify other reasons.

### Statistical analyses

Data from the questionnaire were analyzed using STATA 14.1 (StataCorp, College Station, TX, USA). Respondents were categorized into traditional medicine users and non-users. Data on income, age, BMI, and education were re-classified into appropriate categories. Subjects’ reported income was converted from Nigerian Naira into US Dollars based on the currency exchange rates at the month and year of each subject’s interview. Descriptive statistics were generated and bivariate analyses using Chi-Square and Wilcoxon rank-sum tests were conducted to determine the factors that were significantly associated with TM use. Logistic regression models were then run to identify determinants of TM use among women in the study. Post-estimation tests and trend tests were then conducted to provide information about the global significance of each variable after running the multiple logistic regression. This global test is appropriate before pair-wise comparison. The normal or majority group was used as the referent group in the calculation of odds ratio (OR) and 95% confidence interval (CI).

## Results

### Subject characteristics

Seven hundred fourty-eight adult women were interviewed, with an average age of 42.7 years (range: 18–90 years; SD 14.3). Women reported a median total household monthly income of $94.7 adjusted USD (range: $0–$1594) and a majority had an education level of primary school or below. Most subjects belonged to the Yoruba ethnic group (66.7%), with Hausa (22.3%) and Ibo (4.0%) comprising the largest minority groups. A majority of women reported their occupation as trader (63.8%), with artisan being the second most common occupation (12.2%).

### Usage of traditional medicine

Six hundred women (81.6%) reported having taken TM and 135 (18.4%) reported not having ever taken TM. The reasons for taking TM were varied, with the most commonly reported reasons being fever (40%) and Jedi-jedi/Pile (29%), known as hemorrhoids in the biomedical literature. Table 1 shows the most commonly reported reasons for taking TM. Of those who took TM, only a minority reported taking it everyday (14.3%). Rather, the most frequently reported frequency of use was less than once a week (36.5%), with 31.1% of those women taking TM less than once a week, but more than once a month—the median frequency of usage. Table 2 summarizes the reported frequency of usage for TM users.

**Table 1:** Top Reasons for Taking Traditional Medicine^a^

Reason	Number	Percentage (95% CI)
Fever	340	40.0 (36.8–43.5)
Jedi-jedi/Pile	245	28.9 (25.8–32.0)
Malaria	46	5.41 (3.99–7.15)
Pregnancy	32	3.76 (2.59–5.27)
Stomachache	30	3.53 (2.39–5.00)
Prevention of Disease	20	2.35 (1.44–3.61)
Backache	15	1.76 (0.99–2.89)
Typhoid Fever	15	1.76 (0.99–2.89)
Hypertension	11	1.29 (0.65–2.30)
Dysentery	9	1.05 (0.49–2.00)
Headache	8	0.94 (0.41–1.84)
Diabetes	7	0.82 (0.33–1.69)
Fertility	7	0.82 (0.33–1.69)
Body Ache	6	0.71 (0.26–1.53)
Ulcer	5	0.59 (0.19–1.37)
Body Weakness	4	0.47 (0.13–1.20)
Leg Pain	4	0.47 (0.13–1.20)
Other Diseases	4	0.47 (0.13–1.20)
Menstrual Problems	4	0.47 (0.13–1.20)
Anemia	3	0.35 (0.07–1.03)
Asthma	3	0.35 (0.07–1.03)
Chest Pain	3	0.35 (0.07–1.03)
Diarrhea	3	0.35 (0.07–1.03)
Toothache	3	0.35 (0.07–1.03)
Cold	2	0.24 (0.03–0.85)
Fibroid	2	0.24 (0.03–0.85)
Neck Pain	2	0.24 (0.03–0.85)
Laxative	2	0.24 (0.03–0.85)
Rash	2	0.24 (0.03–0.85)
Cough	2	0.24 (0.03–0.85)
Arthritis	1	0.12 (0.00–0.65)
Breast Pain	1	0.12 (0.00–0.65)
Deworming	1	0.12 (0.00–0.65)
Epilepsy	1	0.12 (0.00–0.65)
Mastitis	1	0.12 (0.00–0.65)
Finger Numbness	1	0.12 (0.00–0.65)
Tumor	1	0.12 (0.00–0.65)
Dizziness	1	0.12 (0.00–0.65)
Throat Pain	1	0.12 (0.00–0.65)
Oily Food Protection	1	0.12 (0.00–0.65)
Well-being	1	0.12 (0.00–0.65)

^a^Total percent does not add up to 100% as some women reported multiple reasons for TM use

**Table 2:** Frequency of Taking Traditional Medicines

Frequency of Usage	Freq.	Percent
Everyday	86	14.3
2–6 times/week	68	11.3
Once a week	46	7.7
< Once a week, but ≥ once a month	99	16.5
<Once a month	219	36.5
Don’t Know	82	13.7
Total	600	100

### Relationship between demographic factors and traditional medicine usage

Table 3 displays demographic characteristics of the women in the study and results of bivariate analyses. Ethnicity, education, occupation, and weight change were statistically significant in the analysis. Women who had been educated were generally less likely to use TM than those who had not had an education, and women who had a secondary education were significantly less likely to use TM than their non-educated counterparts. In addition, women who were traders, the most common occupation, were generally more likely than other occupations to use TM, and significantly so when compared with artisans. The relationship between income and TM usage bordered on significance, and women with the highest total household monthly incomes were more likely to use TM than those with the lowest incomes. No relationship was found between TM usage and age, marital status, or BMI in the bivariate analysis. This lack of significance persisted even after controlling for other demographic factors in a logistic regression.

**Table 3:** Demographics of Traditional Medicine Users and Non-Users

Demographics	TM Users n (%)	Non-Users n (%)	n	***P***-value
Monthly Income				
< $40	129 (75.9%)	41 (24.1%)	671	0.051
$40–$100	160 (84.7%)	29 (15.3%)		
$100–$160	130 (82.8%)	27 (17.2%)		
$160+	132 (85.2%)	23 (14.8%)		
Age Category				
17–29	90 (73.2%)	33 (26.8%)	731	0.13
30–39	194 (84.4%)	36 (15.7%)		
40–49	124 (81.6%)	28 (18.4%)		
50–59	81 (82.7%)	17 (17.3%)		
60+	107 (83.6%)	21 (16.4%)		
Education				
None	116 (88.5%)	15 (11.5%)	688	0.014
Primary	272 (83.7%)	53 (16.3%)		
Secondary	114 (75.0%)	38 (25.0%)		
Vocational/Tech	15 (71.4%)	6 (28.6%)		
College and Above	46 (78.0%)	13 (22.0%)		
Ethnicity				
Yoruba	423 (86.7%)	65 (13.3%)	733	< 0.001
Ibo	16 (55.2%)	13 (44.8%)		
Hausa	122 (73.5%)	44 (26.5%)		
Other	37 (74.0%)	13 (26.0%)		
Occupation				
None	34 (77.3%)	10 (22.7%)	719	0.011
Housewife	44 (78.6%)	12 (21.4%)		
Trader	391 (85.4%)	67 (14.6%)		
Farmer	4 (100%)	0 (0%)		
Artisan	67 (75.3%)	22 (24.7%)		
Professional	35 (81.4%)	8 (18.6%)		
Other	15 (60.0%)	10 (40.0%)		
Current BMI				
Underweight	28 (84.8%)	5 (15.2%)	730	0.93
Normal	221 (81.5%)	50 (18.5%)		
Overweight	180 (80.4%)	44 (19.6%)		
Obese	166 (82.2%)	36 (17.8%)		
Marital Status				
Married	487 (83.4%)	97 (16.6%)	731	0.082
Widowed	81 (75.0%)	27 (25.0%)		
Divorced/Separated	10 (83.3%)	2 (16.7%)		
Never Married	19 (70.4%)	8 (29.6%)		
Weight Change				
Significant Gain	15 (53.6%)	13 (46.4%)	732	< 0.001
Little Gain	100 (74.1%)	35 (25.9%)		
About the same	320 (84.4%)	59 (15.6%)		
Little Loss	144 (85.2%)	25 (14.8%)		
Significant Loss	18 (85.7%)	3 (14.3%)		

Reported *p*-values were calculated from Wilcoxon Rank-Sum tests for Income, Age, Education, BMI, and Weight Change (over the last year) and from Chi-Square tests for Occupation, Marital Status and Ethnicity.

A logistic model, shown in Table 4, was created using significant predictors identified from the bivariate analyses. Income was excluded from the analysis due to maximize sample size and due to the strong correlation between income and ethnicity. Ethnicity, education and weight change remained significant predictors for TM use. Most strikingly, women of Ibo or Hausa were significantly less likely to use TM than women of Yoruba. There was also a positive relationship between TM use and weight loss, with women who reported significant weight loss over the past year significantly more likely to use TM than those whose weight had not changed, and even more so than those who had gained weight. In addition, more educated women were less likely to use TM. Occupation lost statistical significance after controlling for other factors in the logistic regression.

**Table 4:** Multivariable Logistic Regression of Demographic Factors and Traditional Medicine Use (*N* = 668, *p* < 0.001)

Variables	Odds Ratio (95% CI)	*p*-value
Education		
None	1 (ref)	0.039
Primary	0.75 (0.38–1.46)	
Secondary	0.42 (0.21–0.85)	
Vocational/Tech	0.40 (0.12–1.32)	
College and Above	0.53 (0.19–1.49)	
Ethnicity		
Yoruba (ref)	1 (ref)	< 0.001
Ibo	0.25 (0.10–0.63)	
Hausa	0.43 (0.24–0.76)	
Other	0.50 (0.24–1.02)	
Occupation		
Trader (ref)	1 (ref)	0.076
None	0.56 (0.24–1.27)	
Housewife	0.91 (0.40–2.08)	
Farmer	(dropped)	
Artisan	0.55 (0.31–0.97)	
Professional	1.05 (0.37–2.96)	
Other	0.33 (0.13–0.85)	
Weight Change		
Significant gain	0.34 (0.13–0.87)	0.006
Little gain	0.69 (0.40–1.17)	
No change (ref)	1.00 (ref)	
Little loss	1.20 (0.68–2.10)	
Significant loss	1.64 (0.44–6.09)	

*P*-values for testing global association of each variable were generated from post-estimation tests, with the weight change (over the last year) and education variables tested using a trend test

## Discussion

This was the first known study examining the usage of traditional medicine among adult women in Nigeria. Most other studies have focused on TM usage among those with specific illnesses. The rate of usage found in this study, 81.6%, is consistent with that found in other studies of general populations in Nigeria (66.8–84.7%) [1, 25, 26]. The most common reasons for using TM in this study were fever (40%) and Jedi-jedi/pile (29%). Other studies have found malaria to be a common reason, and an herbal preparation known as ‘Agbo jedi-jedi’ to be the most common medicine used [1].

Although this study did not specifically examine TM use in malaria, prior studies have found self-diagnosis and self-medication to be the most common form of diagnosis and treatment for malaria [27–29]. Fevers are often presumed by laypeople to be diagnostic for malaria, which may be problematic because self-diagnoses are more likely to be inaccurate [29]. As two of the top three reasons for using TM were fever and malaria, it is possible that the women in this study were engaging in self-medication without seeking professional medical consultations.

The most striking finding was the inter-ethnic group differences in rates of TM usage. After controlling for other factors, those identifying with the Ibo ethnic group were four times less likely to use traditional medicine than the Yoruba ethnic group (OR = 0.25). Those belonging to the Hausa ethnic group were about half as likely to use traditional medicine than the Yoruba group (OR = 0.43). To our knowledge, this is the first study showing that usage of traditional medicines varied across ethnicities.

As Ibadan, the site of this study, is a majority Yoruba city, individuals from other ethnic groups are most likely immigrants from other areas. This precludes us from generalizing our conclusions to the general Hausa or Ibo populations, as there may be differences between minorities who immigrated to Ibadan and those who stayed in their hometown. In addition, when income is included in the regression model, the significance of ethnicity decreases, suggesting that income differences among the ethnicities may account for some of the differences in TM usage. However, it is important to note that the effect of ethnicity remained significant (*p* = 0.02) even after controlling for factors such as education, income, and occupation. A recent study found that belief in supernatural causes of disease is associated with increased TM usage [20]. It may be possible that beliefs towards health and illness differ among the ethnic groups, thus affecting attitudes and usage of traditional medicine. Now that ethnicity has been identified as a significant determinant, further studies are needed to investigate the reasons behind these differences.

Although past studies have not found a relationship between education level and traditional medicine use [2], our study showed a significant trend towards highly educated women being less likely to use traditional medicine than those with no education, with a significant effect found for women with a secondary education (OR = 0.42). This may be a function of a previously described relationship between education and views on harmful side effects of herbal medicine [21]. Because highly educated women may be more likely to believe that herbal medicine has harmful side effects, they may be less likely to use it. Although previous studies have found a relationship between marital status and traditional medicine use [4], we did not find a significant relationship in our study.

Given that affordability is often cited as a major reason that people use TM rather than biomedicine, we expected to see a relationship between income and TM usage [19]. However, the trend in this study was the opposite, and women with higher total household monthly incomes were more likely to use traditional medicine than those with lower incomes. Although this was not significant, it suggests that affordability may not be the major factor in women’s decision to use TM. It is noteworthy to contrast the inverse relationship between education levels and TM use and the positive relationship between income levels and TM use. Women who are richer are more likely to use TM, as are women who are less educated.

Previous research has found a positive correlation between increasing BMI and herbal medicine use [5]. However, these results were not corroborated in our study, in which current BMI had no significant association with TM use. Interestingly, what did seem to matter was women’s weight change over the past year. Women who gained significant weight over the past year were three times less likely to use traditional medicine (OR = 0.32) when compared to women whose weights did not change. In contrast, the likelihood of using traditional medicine was highest among women who had lost significant weight in the past year (85.7%), and there was a significant association between increasing amounts of weight loss and increased usage of traditional medicine. Women may have experienced weight gain from an improvement in health, resulting in a lower propensity to use traditional medicine. On the other hand, significant weight loss may have been indicative of a major illness, or usage of traditional medicines targeted towards weight loss.

This study had multiple limitations. The questionnaire did not differentiate between herbal medicines and other spiritual or healing practices that may be considered traditional medicine. A further limitation is that we could not determine whether women were taking medicine at the suggestion of an herbal medicine specialist, or if they obtained medicines after self-diagnosis. In addition, there were no questions about the types of medicines used, nor about attitudes and beliefs towards traditional medicine. This limits our ability to draw conclusions about why ethnicity, education, and weight change seem to affect women’s propensity to take traditional medicine. Finally, the community-based recruitment method may have resulted in a sample that is not fully representative of the general population.

The finding that large numbers of women in the general population use traditional medicine is significant for multiple reasons. Chemical analyses of commonly used herbal products have found concerning levels of toxicity and contamination. Given the high rates of traditional medicine use in the general population, it is imperative that more research is done to investigate the safety of these products. In addition, previous studies have found significant associations between traditional medicine use and medication noncompliance [30], but that most patients did not mention traditional medicine use to their doctors [2]. As it is likely that their patients are using traditional medicine, doctors in Nigeria should ask their patients about TM usage as part of the standard interview. Our study only included the healthy controls from the NBCS. However, future studies should examine rates of TM usage among breast cancer patients, and determine whether or not TM usage affects adherence to drug and chemotherapy regimens, as seen in other studies [30].

## Conclusions

In conclusion, our study found a high rate of TM usage among women living in a major urban center in Nigeria consistent with that found in prior studies. This is significant because of the prevalence of contaminated herbal products, the association between TM usage and noncompliance with biomedical treatment regimens. The most striking finding was that ethnicity was significantly correlated with traditional medicine use, with the Ibo and Hausa ethnic groups less likely to use traditional medicine than their Yoruba counterparts. Other departures from previous research include the finding that education was a significant determinant of TM use, and that income was not significant. Future studies should explore the reasons behind differential usage in different ethnic groups, investigate how attitudes and beliefs affect usage rates, and further investigate how TM usage affects medical treatment. TM usage is widespread among the general population in Nigeria, and is likely just as, if not more common among patients with cancer or other illnesses. Given the risks of interference with medication regimens and the prevalence of contaminated herbal products, physicians should regularly engage in conversations with their patients about their TM usage.

## Supplementary information

**Additional file 1.** The original questionnaire used to collect data for this study is included here.

**Additional file 2.** The cover letter for the BMC Complementary Alternative Medicine journal is included here.

## Data Availability

The datasets generated and analyzed during the current study are not publicly available due to the presence of protected health information but are available from the corresponding author on reasonable request.

## Abbreviations

TM: Traditional medicine
OR: Odds ratio
WHO: World Health Organization
CAM: Complementary and alternative medicine
NBCS: Nigerian breast cancer study
UCH: University College Hospital
BMI: Body mass index
CI: Confidence interval
