# Supplementary material for: Traditional medicine usage among adult women in Ibadan, Nigeria: a cross-sectional study
Source: BMC Complement Med Ther. 2020 Mar 20;20:93. doi: 10.1186/s12906-020-02881-z (PMC7083039; doi:10.1186/s12906-020-02881-z)
Supplement: Supplementary file 1 — Additional file 1. The original questionnaire used to collect data for this study is included here. [file 12906_2020_2881_MOESM1_ESM.docx]

Dear Anne Menard,

Thank you for enclosing the reviewers’ comments and for the time you have taken to review our research paper, “Traditional Medicine Usage among Adult Women in Ibadan, Nigeria: A Cross-sectional Study” (BCAM-D-19-00939R1). We have carefully reviewed the comments and have revised the manuscript accordingly. Our responses are given in a point-by-point manner below. Changes to the manuscript are shown through the track changes function.

We hope the revised version is now suitable for publication and look forward to hearing from you in due course.

Sincerely,

Suellen Li, MD
Department of Internal Medicine

Massachusetts General Hospital

Editor Comments:
1. Please change the Materials and Methods heading to Methods.

**This change has now been made.**

2. Please clarify in the Ethics approval and consent to participate section if the informed consent was written or verbal. If verbal, why was this method chosen and was this approved by the ethics committee?

**We have now clarified that written consent was obtained in the Ethics approval and consent to participate section.**

3. Please add a section "Additional files" (after the References/Figure legends) where you list the following information for each additional/supplementary file in the file inventory:
- File name (e.g. Additional file 1)
- Title of data
- Description of data

**This section has now been added at the end of the References/Figure legends.**

Lidiane Albuquerque (Reviewer 1): - I suggest only updating references 8, 9 and 18.

**We have now updated references 8, 9, and 18 and correspondingly updated the information in the paper (the ratio of traditional healers to population compared to that of medical doctors to population has now been updated from 80 to 100).**

**Thank you for noticing the omission of Reference 14 in the references list – we have now included it in the article.**

Muhammad al-Attar, M.D.; Ph.D (Reviewer 2):

Using complementary, alternative or traditional medicine are common around all the world, studies in these topics are sufficient; Publishing local epidemiological studies in international journals is not in priority anymore.
Besides, there are some issues which should be addressed before the publication of this paper in another journal:

**We appreciate you bringing up this concern about the suitability of publishing this paper in *BMC Complementary and Alternative Medicine*. However, previous studies have documented that Nigerian immigrants often bring their health beliefs and practices to their new countries (**[**https://www.ncbi.nlm.nih.gov/pubmed/22924201**](https://www.ncbi.nlm.nih.gov/pubmed/22924201)**). As a result, we thought it was suitable for publication in an international journal of complementary and alternative medicine to allow medical providers across the world to familiarize themselves with the practices of their Nigerian patients. In addition, multiple studies of the traditional medicine usage in Nigeria have been published in *BMC Complementary and Alternative Medicine*, which is why we thought it would be a suitable fit for our findings. The below is a list from our references section of similar studies, all of which have smaller sample sizes and more niche populations, that have been published in this journal:**Oreagba, I., et. al. Herbal medicine use among urban residents in Lagos, Nigeria. *BMC Complementary and Alternative Medicine*. 2011;11:117. (n=388)

Ezeome, E., Anarado, A. Use of complementary and alternative medicine by cancer patients at the University of Nigeria Teaching Hospital, Enugu, Nigeria. *BMC Complementary and Alternative Medicine*. 2007;7:28. (n=160)

Amira, O., Okubadejo, N. Frequency of complementary and alternative medicine utilization in hypertensive patients attending an urban tertiary care centre in Nigeria. *BMC Complementary and Alternative Medicine*. 2007;7:30. (n=225)

Oshikoya, K., et. al. Use of complementary and alternative medicines for children with chronic health conditions in Lagos, Nigeria. *BMC Complementary and Alternative Medicine*. 2008;8:66. (n=318)

Fakeye, T., Adisa, R., Musa, I. Attitude and use of herbal medicines among pregnant women in Nigeria. *BMC Complementary and Alternative Medicine*. 2009;9:53. (n=595)

Onyiapat, J., Okoronkwo, I., Ogbonnaya, N. Complementary and alternative medicine use among adults in Enugu, Nigeria. *BMC Complementary and Alternative Medicine*. 2011;11:19. (n=732).

1) The article is still in review by authors, many «track changes» are still active, without accepting or reject!

**Per the request of the journal, we have ensured that all new changes to the manuscript are indicated in the text using the track changes function.**

2) «Questionnaire» is a scientific term, its designing go through a logic process, this article mentioned it just as «A structured questionnaire was created» without any information about its validity or reliability!
Furthermore, it seems that some data collected by interviewing checklist. So, the original Questionnaire and/ or checklist should be attached to the article.

**We have attached the original questionnaire to our submission. Unfortunately, there are no standardized traditional medicine questionnaires for the Nigerian context. As a result, we initiated the creation of our own questionnaire and standardized our interviewers to ensure consistency of response.**


leila rezaei (Reviewer 3): No comment.


Malcolm Koo, PhD (Reviewer 4): The manuscript reported the results of a cross-sectional study on the factors associated with traditional medicine usage among adult women in Ibadan, Nigeria.
The article is generally well-written and structured but a number of clarifications should be addressed.

Major comments:

1. Methods, page 8, line29: Reproductive history and hormone use were ascertained in the questionnaire, but they were not included in Table 3 and 4. Please explain why.

**We found that use of hormones was too rare in our population to be able to conduct a meaningful analysis. We have now removed mention of reproductive history and hormone use in the methods section to avoid confusion.**

2. Methods, page 9, line 18: Why did a variable selection procedure, such as stepwise, not used in the multiple logistic regression analysis.

**We agree that there are pros and cons of stepwise selection procedure. Some leading statisticians do not favor stepwise selection (for example,** <https://www.stata.com/support/faqs/statistics/stepwise-regression-problems/>**). A formal automatic stepwise selection may be used when sample size and predictor pools are large and the purpose of the analysis is for prediction. We ultimately chose to use a bivariate screening procedure to purposely select variables based on our knowledge of the field and because we do not have a long list of variables. Then we built a final logistic model using an informal forward selection approach.**

3. Methods: Previous literature suggested that marital status could affect the use of complementary medicine, why did the authors omit marital status from their analysis?

**We appreciate you bringing up this observation. We have now included our analysis on marital status in Table 3, which shows a nonsignificant relationship between marital status and use of complementary medicine. We have included commentary on this lack of relationship in our manuscript and contrasted it to previous literature finding that marital status could affect the use of complementary medicine and included this in the 5^th^ paragraph of the Discussion Section.**

4. Methods, page 9, line 20: Readers will benefit if the reasons for using post-estimation tests and trend tests are provided. I noted that the authors did previously reply to another reviewer who had a similar comment. The authors wrote that "This practice is quite common for continuous outcome using ANOVA and less for binary outcome using logistic regression, but the same good statistical practice (global test followed by pair-wise tests) should be used regardless of data type." A shortened version of this description should be included in the statistical analysis section, and preferably with a reference to support this less common practice.

**Thank you for this suggestion. We have now included a shortened version of this description in the statistical analysis section.**

5. Methods, Results, and tables: Looking at the questionnaire, "monthly income" should be "total household monthly income". Please revise throughout the manuscript accordingly.

**We have** **now revised the term “monthly income” to be “total household monthly income.”**

6. Methods: Please explain why monthly income (borderline significant) was not included in the multiple regression analysis? The authors have provided an explanation in the Discussion that "when income is included in the regression model, the significance of ethnicity decreases, suggesting that income differences among the ethnicities may account for some of the differences in TM usage. (page 16, line 22)".
However, the authors did not provide further analysis to demonstrate that income differences and ethnicities are indeed strongly correlated. For example, if multicollinearity between income and ethnicities indeed exists, it can be a valid reason for not including income in the regression model.

**We decided to exclude income from the analysis because it was not significant in the bivariate analysis. Furthermore, including income in the multiple regression analysis would have resulted in a significant reduction in the sample size because it was the main variable that received the fewest responses in the survey. We conducted a test of the relationship between the monthly income and ethnicity, and found a strong association between the two variables (see the below table). In addition, income and education was strongly correlated. For these reasons, we decided to exclude income from the multiple variable regression model. We have now noted these reasons in the manuscript.**

Relationship between Ethnicity and Monthly Household Income

|  | **Monthly Income** |  |  |  |
| --- | --- | --- | --- | --- |
| **Ethnicity** | <$40 | $40-$100 | $100-$160 | $160+ |
| Yoruba | 63 (13.7%) | 143 (31.0%) | 134 (29.1%) | 121 (26.2%) |
| Ibo | 3 (11.5%) | 10 (38.5%) | 3 (11.5%) | 10 (38.5%) |
| Hausa | 99 (68.3%) | 27 (18.6%) | 11 (7.6%) | 8 (5.5%) |
| Other | 8 (17.4%) | 10 (21.7%) | 12 26.1%) | 16 (34.8%) |

**Chi-2 190.5016; p<0.001**

7. Discussion, page 16, line 44: The authors indicated that "highly educated women were less likely to use traditional medicine than those with no education, with a significant effect found for women with a secondary education (OR = 0.42)." The first part of the sentence is not entirely correct given that the odds ratios were not significant for women with an educational level of vocational/tech and college and above. Perhaps this was an issue of statistical power, which may be alleviated if the two groups were collapsed into a group "vocational/technical school, college, and above".

**Thank you for this astute suggestion. We re-ran the logistic regression after combining the vocational/technical school with college and above but that group remained not significant with an OR of 0.48 (0.19-1.18). As a result, decided to keep the original categorization of educational groups. We have revised the first part of the sentence to mention that the trend test was significant for a higher level of education correlating with lower use of traditional medicine.**

8. Discussion, page 17, line 24: The authors indicated that "Women who are richer, but less educated are more likely to use TM." and this postulation may potentially be tested statistically by creating a new composite variable (high income, less educated; low income, less educated; high income, more educated; low income, more educated) with dichotomized income and educational levels.

**Thank you for the comment. We had previously conducted analyses looking exactly as suggested with dichotomized income and educational levels. Because income was significantly positively correlated with education level (p<0.001), there was a relative rarity of combinations of high income/less educated and less income/more educated. We did not find our results compelling enough to include in the final manuscript. We have revised the discussion as follows, “Women who are richer are more likely to use TM, as are women who are less educated.”**

9. Discussion, page 17, line 33: The authors should provide some possible reasons for the observation that "Women who gained significant weight over the past year were three times less likely to use traditional medicine (OR = 0.32) when compared to women whose weights did not change."
**Thank you for the suggestion. We postulate that weight change is an indicator of health status. The weight gain may indicate better health, resulting in a lower propensity to use traditional medicine. In contrast, weight loss indicates worsening in health, and women with worsening in health use more traditional medicine. We have now included this in the 7^th^ paragraph of our Discussion section.**

10. Discussion, page 17, line 37: The authors indicated that "The likelihood of using traditional medicine was highest among women who had lost significant weight in the past year (85.7%)". I think this statement may be misleading. This is only the result obtained from the bivariate analysis, but this association was not significant in the multiple regression model.

**Thank you for pointing out how that sentence may be misleading – the association for women who had lost significant weight in the last year was indeed not significant in the multiple regression. We have now clarified in that sentence that it was the overall trend for weight loss that was significant in the trend test of the multiple regression model.**

11. Conclusion, page 19, line 12, 25, and 27: Since the participants of this study were healthy individuals, the mentioning of "chemotherapy" and "cancer treatment" should be removed.

**We have now removed mention of chemotherapy and cancer treatment from the conclusion section.**

12. Table 1: Please provide 95% CI for the percentages.

**The 95% CI percentages have now been added for Table 1.**

13. Table 3 and 4: Please consider changing the variable "weight change" to "weight change over the past year" or specify its meaning in the footnotes.
**We have now included a statement in the methods section under “Questionnaire” to specify the meaning of the weight change variable. We have also specified the meaning of the weight change variable in the footnotes of Table 3 and Table 4.**

Minor comments:

1. Table 4: It is redundant to include Chi-square value when exact p value is provided. In addition, P=0.0055 should be presented as 0.006 for consistency.

**We have now removed the Chi-square value column from the table and have changed-0.0055 to 0.006.**

2. Page 22, Line 32: Sentence case should be used.
**We have now revised our references so that they all consistently use sentence case.**

3. Page 6, Line 20: A reference (no. 15) should be placed immediately after the relevant sentence.

**On Page 6, Line 20, the relevant reference is listed immediately after the sentence.**

4. Page 22, Line 21: Reference no. 14 is missing from the References section, but it was cited in the text (page 6, line 24). Please revise the references section.

**Thank you for your close attention to our paper and references. We have now revised the references section to include reference number 14, which had been mistakenly omitted from our paper.**

5. Page 21: Abbreviated journal title should be used instead of full title for Reference no. 1, 2, 4, 5, 6, 10, 15, 16, 18, 20, 21, 25, 26, 28, 29, and 30.
**Thank you for pointing this out and taking the time to list the specific references – the abbreviated journal titles are now used for all of the references.**
