# Supplementary material for: Traditional medicine usage among adult women in Ibadan, Nigeria: a cross-sectional study
Source: BMC Complement Med Ther. 2020 Mar 20;20:93. doi: 10.1186/s12906-020-02881-z (PMC7083039; doi:10.1186/s12906-020-02881-z)
Supplement: Supplementary file 2 — Additional file 2. The cover letter for the BMC Complementary Alternative Medicine journal is included here. [file 12906_2020_2881_MOESM2_ESM.doc]

Place bar code here

# Nigerian Breast Cancer Study

Epidemiologic Questionnaire

DATE OF INTERVIEW:	__ __ / __ __ __ / __ __ __ __

DAY	MONTH	YEAR

*(****Record month in 3-letter abbreviations like JAN, FEB, MAR,…DEC****)*

DATE OF DIAGNOSIS:	__ __ / __ __ __ / __ __ __ __

DAY	MONTH	YEAR

*(****Record month in 3-letter abbreviations like JAN, FEB, MAR,…DEC****)*

CASE/CONTROL STATUS:	Case	Control

# I NTERVIEWER’S NAME: _____________________________

SUPERVISOR’S NAME**:** ___________________________________________

##	*Please print name clearly*

**SECTION A. BACKGROUND INFORMATION**

*I would like to begin by asking for some background information about you and your family*.

A1.	What is your date of birth?	__ __ / __ __ __ / __ __ __ __

DAY	MONTH	YEAR

*(****Record month in 3-letter abbreviations like JAN, FEB, MAR,…DEC****)*

A1a. What is your current age	____

A2.	Sex:	Male – 1; Female – 2	____

A3.	What was the highest level of education you completed?

None	0

Primary	1

Secondary	2

Vocational/Technical	3

Polytechnic/OND/Some college	4

Bachelor's degree/HND	5

Postgraduate degree	6

Don't know	9

Other (Please Specify)	7

____________

A4.	Marital Status.

Married	1

Widowed	2

Divorced	3

Separated	4

Never married	5

Don't Know	9

A5.	Occupation?

None	0

Housewife	1

Trader	2

Farmer	3

Artisan	4

Professional	5

Don't know	9

OTHER **(SPECIFY)**	8

A6.	What is your total household monthly income? ________ Naira

A7	Do you own … ?

|__| a radio |__| a bicycle |__| a motorcycle |__| a car

|__| a cell phone |__| a television |__| none of above |__| refused to answer

A9.	Which of the following religions do you currently practice?

Christian	1

Muslim	2

None	3

Don't know	9

OTHER **(SPECIFY)**	8

A10.	Which of the following choices best describes your ethnic background?

Yoruba	1

Ibo	2

Hausa	3

Don't know	9

OTHER **(SPECIFY)**	8

A10A.	Which of the following choices best describes your mother's ethnic background?

Yoruba	1

Ibo	2

Hausa	3

Don't know	9

OTHER **(SPECIFY)**	8	__________________

A10B.	Which of the following choices best describes your father's ethnic background?

Yoruba	1

Ibo	2

Hausa	3

Don't know	9

OTHER **(SPECIFY)**	8	__________________

**SECTION B. MEDICAL HISTORY**

*The next section asks questions about some illnesses you may have had*.

B1.	Have you had a previous history of cancer?

YES	1

NO	2	**GO TO B5.**

Don't Know	9	**GO TO B5**.

**IF YES:**

|  | CANCER #1 | CANCER #2 | CANCER #3 |
| --- | --- | --- | --- |
| B2. What type of cancer did you have?  Breast 1  Ovarian 2  Cervix 3  Leukemia 4  Don’t know 9  Other 8 | ____  _______________ | ____  _______________ | ____  _______________ |
| B3. How old were you when this cancer was first diagnosed?  IF UNKNOWN, CODE 99 | AGE __ __ | AGE __ __ | AGE __ __ |
| B4. In what year were you diagnosed with this cancer? |  |  |  |

B5.	Has a doctor ever told you that you had benign breast disease, such as a non-cancerous cyst or a breast lump? (not including the last year)

YES	1

NO	2	**GO TO B7.**

Don't Know	9	**GO TO B7.**

**IF YES:**

B6.	What was the doctor’s diagnosis?

| a. Diagnosis | b. Age | c. left right both unknown | d. Removed by surgery?  Yes No |
| --- | --- | --- | --- |
| 1. Breast Cyst | __ __ | 1 2 3 9 | 1 2 |
| 2. Fibroadenoma | __ __ | 1 2 3 9 | 1 2 |
| 3. Intraductal Papilloma | __ __ | 1 2 3 9 | 1 2 |
| 4. Atypical Hyperplasia | __ __ | 1 2 3 9 | 1 2 |
| 9. Don’t Know | **__ __** | 1 2 3 9 | 1 2 |
| 8. Other  _________________ | **__ __** | 1 2 3 9 | 1 2 |

**IF AGE UNKNOWN**, CODE AS 99.

B7.	Have you ever been treated for an ovarian problem or had surgical removal of a pelvic mass that was not cancer?

YES	1

NO	2	**GO TO B9.**

Don't Know	9	**GO TO B9.**

**IF YES:**

B8.	What was the doctor’s diagnosis? (where a pathology report was obtained)

| Diagnosis | b. Age at diagnoses | c. left right both unknown | d. Removed by surgery?  Yes No |
| --- | --- | --- | --- |
| 1. Ovarian cyst | __ __ | 1 2 3 9 | 1 2 |
| 2. Don’t Know | __ __ | 1 2 3 9 | 1 2 |
| 3. Other  ___________________ | __ __ | 1 2 3 9 | 1 2 |

**IF AGE UNKNOWN**, CODE AS 99.

B9. Has a doctor ever said that you have HIV or AIDS?

YES	1

B9a. At what age were you first told this? __ __ years

NO	2

Don't Know	9

B10. Has a doctor ever said that you have diabetes (high sugar in blood or urine)?

B10a. At what age were you first told this? __ __ years

B10b. Was this during pregnancy only?

YES	1

NO	2

Don’t Know	9

B10c. Have you ever been treated for your diabetes?

Insulin	1

Pills	2

Diet	3

No treatment	4

Don’t Know	9

B10d.	What type of diabetes do you have?

Type 1	1

Type 2	2

Don’t know	9

YES	1

NO	2

Don't Know	9

B11. Are you currently taking NSAID medications (such as Aspirin, Indocid, Alabukun, Ibuprofen, Feldene, Cataflam, Naproxen, and Phensic) at least three times a week (do not include Panadol, Tylenol, Tramadol or other non-NSAID pain relievers)?

B11a. Name of the medication _____________

B11b. How many tablets/capsules are you taking?

B11b1 __ __ per day

**OR**

B11b2 __ __ per week

B11c. How long have you taken this medication?

__ __	__ __

Years Months

YES	1

NO	2

Don't Know	9

**SECTION C. SURGERIES AND MAMMOGRAMS**

Now I have some questions about surgeries you may have had.

C1.	Up until one year before your diagnosis of cancer (or one year before this interview), did you ever have a breast completely removed?

YES	1

NO	2	**GO TO C5.**

Don't Know	9	**GO TO C5.**

**IF YES:**

C2.	Did you have your right or left breast removed?

RIGHT ONLY	1

LEFT ONLY	2

BOTH	3

C3.	How old were you when you had your breast(s) removed?

RIGHT ONLY	___ ___

LEFT ONLY	___ ___

BOTH	___ ___

**IF UNKNOWN**, CODE AS 99.

C4.	Why was your breast(s) surgically removed?

Because of breast cancer or a

suspicious lump in that breast	1

To prevent the development

of breast cancer	2

Don't Know	9

OTHER (**SPECIFY**)	8

_________________________

C5.	For cases: Is this diagnosis of cancer a recurrence of breast cancer you had before?

YES	1

NO	2

Don't Know	9

C6.	A breast biopsy is the removal of breast tissue by surgery for the purpose of making a diagnosis. Have you ever had a breast biopsy or lumpectomy that was diagnosed as cancer? Please do not include fine needle biopsy.

YES	1

NO	2	**GO TO C8.**

Don't Know	9	**GO TO C8.**

**IF YES:**

C7.	How old were you when this was first done?	___ ___

**IF UNKNOWN**, CODE AS 99.

C8.	Have you ever had a breast biopsy that was diagnosed as benign breast disease, such as a non-cancerous cyst or a breast lump? Please do not include fine needle biopsy.

YES	1

NO	2	**GO TO C10.**

Don't Know	9	**GO TO C10.**

**IF YES:**

C9.	How old were you when this was first done?	___ ___

**IF UNKNOWN**, CODE AS 99.

C10.	Up until one year before your diagnosis of cancer (or one year before this interview), did you ever have an ovary completely removed?

YES	1

NO	2	**GO TO C14.**

Don't Know	9	**GO TO C14.**

**IF YES:**

C11.	Did you have one or both ovaries removed?

ONE	1

BOTH	2

Don't Know	9

C12.	How old were you when you had your ovary(ies) removed?

LEFT OVARY	___ ___

RIGHT OVARY	___ ___

BOTH	___ ___

**IF UNKNOWN**, CODE AS 99.

C13.	Why was your ovary(ies) surgically removed?

Because of ovarian cancer	1

To prevent the development

of ovarian cancer	2

Don't Know	9

OTHER (**SPECIFY**)	8

_______________________________

*Now I have some questions about mammograms*.

C14.	A mammogram is an x-ray examination of the breasts.
Have you ever had a mammogram?

YES	1

NO	2	**GO TO D1.**

Don't Know	9	**GO TO D1.**

**IF YES:**

C15.	How old were you when you had your first mammogram?	__ __

**IF UNKNOWN**, CODE AS 99.

C16.	When did you have your most recent mammogram?

__ __ / __ __ __ / __ __ __ __

DAY	MONTH	YEAR

*(****Record month in 3-letter abbreviations like JAN, FEB, MAR,…DEC****)*

**IF UNKNOWN**, CODE AS 99/XXX/9999

**SECTION D. HEIGHT AND WEIGHT**

*Now we will need to get clinical measurements from you*

D1.	Height	__ __ __ (cm)

D3.	Waist	__ __ __ (kg)

D2.	Weight	__ __ __ (kg)

D4.	Hip	__ __ __ (cm)

D5.	Compared to your current weight, would you say your weight one year before your diagnosis (or one year before the interview) was:

Significantly less than now	1

A little less than now	2

About the same as now	3

A little more than now	4

Significantly more than now	5

*Please look at below silhouettes and answer questions D6-D9:*

1	2	3	4	5	6	7	8	9

D6.	Which silhouette best represents your current body image?

Silhouette # __ __

D7.	Which silhouette best represents your body image at age 18?

Silhouette # __ __

D8.	Which silhouette best represents your body image at age 30?

Silhouette # __ __

D9.	Which silhouette best represents your body image at age 40?

Silhouette # __ __

**SECTION E. ALCOHOL**

*Now I will be asking you about alcoholic beverages you may have consumed.*

E1.	Have you ever consumed any alcoholic beverages, such as beer, wine, or local gin at

least once a week for 6 months or longer?

YES	1

NO	2	**GO TO G1.**

Don't Know	9	**GO TO G1.**

**IF YES:**

E2.	At what age did you first start consuming alcoholic beverages at least

once a week for 6 months or longer?

AGE __ __

**IF UNKNOWN**, CODE AS 99.

E3.	Up until one year before your diagnosis of cancer (or one year before this interview), were you consuming alcoholic beverages at least once a week?

YES	1	**GO TO E5.**

NO	2

Don't Know	9	**GO TO E5.**

**IF NO:**

E4.	At what age did you stop consuming alcoholic beverages at least

once a week?

AGE __ __

E5.	Up until one year before your diagnosis of cancer (or one year before this interview), for how many years in total did you consume alcoholic beverages at least once a week?

YEARS __ __

**IF UNKNOWN**, CODE AS 99.

E6.	Up until one year before your diagnosis of cancer (or one year before this interview), when you consumed alcoholic beverages at least once a week, how many shots (5 cl), glasses (15 cl) or bottles (about 35 cl) did you usually have in a week?

1. Local gin	__ __ Glasses OR __ __ Shots

2. Imported gin/brandy	__ __ Glasses OR __ __ Shots

3. Palm wine	__ __ Glasses OR __ __ Bottles

4. Imported wine	__ __ Glasses OR __ __ Bottles

5. Beer	__ __ Glasses OR __ __ Bottles

**SECTION G. GYNAECOLOGICAL HISTORY**

*The following questions are about menstruation and the use of birth control pills and other hormonal contraceptives*.

G1.	At what age did you develop breast buds?	AGE __ __

G2.	Have you ever had a menstrual period?

YES	1

NO	2	**GO TO G6.**

Don't Know	9	**GO TO G6.**

**IF YES:**

G3.	At what age did you have your first menstrual period?	AGE __ __

**IF UNKNOWN**, CODE AS 99.

G4.	Have you had regular periods for the most time of your life? (excluding times of pregnancy, breast feeding, or contraceptive use.)

YES	1

NO	2

Sometimes	8

G5	Excluding the time when you were using contraceptives, what is the length of your usual menstrual cycle (i.e. From the first day of one period to the first day of the next period.)?

__ __ days

G6.	Primary amenorrhea is the failure of menstrual periods to start

naturally. Has a doctor ever told you that you had primary amenorrhea?

YES	1

NO	2	**GO TO G8.**

Don't Know	9	**GO TO G8.**

**IF YES:**

G7.	How old were you when this was first diagnosed?	AGE __ __

**IF UNKNOWN**, CODE AS 99.

G8.	Have you ever used hormonal contraceptives, in the form of birth control pills,

implants, or injections?

YES	1

NO	2	**GO TO H1.**

Don't Know	9	**GO TO H1.**

**IF YES:**

G9.	What type of contraceptives did you use?

Pill	1

Injection	2

Implant	3

Other	8

Other Specify______________

Don’t know	9

| G10. Name of the hormonal contraceptive. | G11. Age of first use. | G12. Cumulative duration  of use. | G13. Age of last use |
| --- | --- | --- | --- |
| Pill  Yes 1  No 2  Don’t Know 9 | __ __ | Month 1  Year 2 __ __ | __ __ |
| Injection  Yes 1  No 2  Don’t Know 9 | __ __ | Month 1  Year 2 __ __ | __ __ |
| Implant  Yes 1  No 2  Don’t Know 9 | __ __ | Month 1  Year 2 __ __ | __ __ |
| Other ________  Yes 1  No 2  Don’t Know 9 | __ __ | Month 1  Year 2 __ __ | __ __ |

G14.	Are you currently taking hormonal contraceptives?

YES	1

NO	2

Don't Know	9

G15.	Up until one year before your diagnosis of cancer, for how many years

in total did you take hormonal contraceptives?

**IF LESS THAN 1 YEAR:**	CODE 00	YEARS __ __

**IF UNKNOWN**, CODE AS 99.

**SECTION H. PREGNANCY HISTORY**

*Now I’ll be asking about your pregnancy history. Please include all live births, miscarriages, stillbirths, and other outcomes.*

H1.	Have you ever been pregnant?

YES	1

NO	2	**GO TO H13.**

Don't Know	9	**GO TO H13.**

**IF YES:**

H2.	How many pregnancies have you had?	__ __

H3.	How many live births have you had?	__ __

**IF NO LIVE BIRTHS, GO TO INTRODUCTION AFTER H6**.

H4.	How old were you when you had your first live birth?	AGE	__ __

**IF UNKNOWN**, CODE AS 99.

H5.	What is the date of your last live birth?	__ __ / __ __ __ / __ __ __ __

DAY	MONTH	YEAR

*(****Record month in 3-letter abbreviations like JAN, FEB, MAR,…DEC****)*

H6.	Did you ever breast-feed a child for one month or longer?

YES	1

NO	2

Don't Know	9

*Now I would like to ask you about each pregnancy you have had.*

|  | 1ST  PREGNANCY | 2ND PREGNANCY | 3RD  PREGNANCY |
| --- | --- | --- | --- |
| H7. What was the outcome of your pregnancy?  Currently pregnant 1  Single live birth 2  Multiple birth 3  Stillbirth 4  Miscarriage 5  Tubal or ectopic pregnancy 6  Induced abortion 7  Don’t know 9 | _____ | _____ | _____ |
| H8. During what month and year (was your baby born / did this pregnancy end)? | __ __ /__ __ __ _  MO YEAR | __ __ /__ __ __ _  MO YEAR | __ __ /__ __ __  MO YEAR |
| H9.How long was this pregnancy (in months)? | __ __  # of months | __ __  # of months | __ __  # of months |
| **IF SINGLE LIVE BIRTH OR STILLBIRTH:**  H10. Did you have a boy or a girl?  **IF MULTIPLE LIVE BIRTHS OR STILLBIRTHS:**  H10. How many boys and girls did you have?  **IF UNKNOWN**, CODE AS 99. | ____  # of BOYS    ____  # of GIRLS | ____  # of BOYS    ____  # of GIRLS | ____  # of BOYS    ____  # of GIRLS |
| **IF LIVE BIRTH(S):**  H11. Did you breast-feed (this child / these children)?  **IF YES:**  H12. For how many months did you breast-feed (this child / these children)? | YES 1  NO 2  Don’t know 9    __ __  # of months | YES 1  NO 2  Don’t know 9    __ __  # of months | YES 1  NO 2  Don’t know 9  __ __  # of months |

|  | 4TH PREGNANCY | 5TH PREGNANCY | 6TH  PREGNANCY |
| --- | --- | --- | --- |
| H7. What was the outcome of your pregnancy?  Currently pregnant 1  Single live birth 2  Multiple birth 3  Stillbirth 4  Miscarriage 5  Tubal or ectopic pregnancy 6  Induced abortion 7  Don’t know 9 | _____ | _____ | _____ |
| H8. During what month and year (was your baby born / did this pregnancy end)? | __ __ /__ __ __ _  MO YEAR | __ __ /__ __ __ _  MO YEAR | __ __ /__ __ __  MO YEAR |
| H9.How long was this pregnancy (in months)? | __ __  # of months | __ __  # of months | __ __  # of months |
| **IF SINGLE LIVE BIRTH OR STILLBIRTH:**  H10. Did you have a boy or a girl?  **IF MULTIPLE LIVE BIRTHS OR STILLBIRTHS:**  H10. How many boys and girls did you have?  **IF UNKNOWN**, CODE AS 99. | ____  # of BOYS    ____  # of GIRLS | ____  # of BOYS    ____  # of GIRLS | ____  # of BOYS    ____  # of GIRLS |
| **IF LIVE BIRTH(S):**  H11. Did you breast-feed (this child / these children)?  **IF YES:**  H12. For how many months did you breast-feed (this child / these children)? | YES 1  NO 2  Don’t know 9    __ __  # of months | YES 1  NO 2  Don’t know 9    __ __  # of months | YES 1  NO 2  Don’t know 9  __ __  # of months |

|  | 7TH PREGNANCY | 8TH PREGNANCY | 9TH  PREGNANCY |
| --- | --- | --- | --- |
| H7. What was the outcome of your pregnancy?  Currently pregnant 1  Single live birth 2  Multiple birth 3  Stillbirth 4  Miscarriage 5  Tubal or ectopic pregnancy 6  Induced abortion 7  Don’t know 9 | _____ | _____ | _____ |
| H8. During what month and year (was your baby born / did this pregnancy end)? | __ __ /__ __ __ _  MO YEAR | __ __ /__ __ __ _  MO YEAR | __ __ /__ __ __  MO YEAR |
| H9.How long was this pregnancy (in months)? | __ __  # of months | __ __  # of months | __ __  # of months |
| **IF SINGLE LIVE BIRTH OR STILLBIRTH:**  H10. Did you have a boy or a girl?  **IF MULTIPLE LIVE BIRTHS OR STILLBIRTHS:**  H10. How many boys and girls did you have?  **IF UNKNOWN**, CODE AS 99. | ____  # of BOYS    ____  # of GIRLS | ____  # of BOYS    ____  # of GIRLS | ____  # of BOYS    ____  # of GIRLS |
| **IF LIVE BIRTH(S):**  H11. Did you breast-feed (this child / these children)?  **IF YES:**  H12. For how many months did you breast-feed (this child / these children)? | YES 1  NO 2  Don’t know 9    __ __  # of months | YES 1  NO 2  Don’t know 9    __ __  # of months | YES 1  NO 2  Don’t know 9  __ __  # of months |

|  | 10TH PREGNANCY | 11TH PREGNANCY | 12TH  PREGNANCY |
| --- | --- | --- | --- |
| H7. What was the outcome of your pregnancy?  Currently pregnant 1  Single live birth 2  Multiple birth 3  Stillbirth 4  Miscarriage 5  Tubal or ectopic pregnancy 6  Induced abortion 7  Don’t know 9 | _____ | _____ | _____ |
| H8. During what month and year (was your baby born / did this pregnancy end)? | __ __ /__ __ __ _  MO YEAR | __ __ /__ __ __ _  MO YEAR | __ __ /__ __ __  MO YEAR |
| H9.How long was this pregnancy (in months)? | __ __  # of months | __ __  # of months | __ __  # of months |
| **IF SINGLE LIVE BIRTH OR STILLBIRTH:**  H10. Did you have a boy or a girl?  **IF MULTIPLE LIVE BIRTHS OR STILLBIRTHS:**  H10. How many boys and girls did you have?  **IF UNKNOWN**, CODE AS 99. | ____  # of BOYS    ____  # of GIRLS | ____  # of BOYS    ____  # of GIRLS | ____  # of BOYS    ____  # of GIRLS |
| **IF LIVE BIRTH(S):**  H11. Did you breast-feed (this child / these children)?  **IF YES:**  H12. For how many months did you breast-feed (this child / these children)? | YES 1  NO 2  Don’t know 9    __ __  # of months | YES 1  NO 2  Don’t know 9    __ __  # of months | YES 1  NO 2  Don’t know 9  __ __  # of months |

CONTINUATION PAGE USED:	YES	1

NO	2

H13.	Have you ever taken a drug for infertility to try to become pregnant?

YES	1

NO	2	**GO TO J1.**

Don’t know	9	**GO TO J1.**

**IF YES:**

H14.	Which of the following drugs did you take? **(CIRCLE AS MANY AS APPLY)**

YES	NO	Don’t know

Clomid	1	2	9

Pergonal	1	2	9

Serophene	1	2	9

hCG	1	2	9

Metrodin	1	2	9

Other	1	2	9

**(SPECIFY)**

__________________________________

| Name of the Fertility Medication | H15. Age of first use. | H16. Cumulative duration of use. | H17. Age of last use |
| --- | --- | --- | --- |
| Clomid | __ __ | Month 1  Year 2 __ __ __ | __ __ |
| Pergonal | __ __ | Month 1  Year 2 __ __ __ | __ __ |
| Serophene | __ __ | Month 1  Year 2 __ __ __ | __ __ |
| hCG | __ __ | Month 1  Year 2 __ __ __ | __ __ |
| Metrodin | __ __ | Month 1  Year 2 __ __ __ | __ __ |
| Other _______________ | __ __ | Month 1  Year 2 __ __ __ | __ __ |

**IF UNKNOWN**, CODE AS 99.

H18.	Was the drug prescribed for infertility as part of GIFT, which means gamete intra-fallopian transfer, or as part of IVF, which means in vitro fertilization or as part of IUI (Intrauterine insemination) or as part of ICSI (Intracytoplasmic sperm injection)?

YES	1

NO	2

Don’t know	9

**SECTION J: MENOPAUSE**

*The next section asks questions about your menstrual history and use of menopausal hormones.*

J1.	How long ago was your last menstrual period?

Less than 1 month	1

1 to 6 months	2

7 to 11 months	3

1 year or more	4

Never had a period	5	**GO TO K1.**

Don’t know	9

J2.	Have your menstrual periods stopped for 1 year or more? Please do not include

times when your periods stopped because of pregnancy, breast-feeding, serious illness or strenuous exercise.

YES	1	**GO TO J3**

NO	2	**GO TO K1.**

Don’t know	9	**GO TO K1.**

J3.	How old were you when you had your last menstrual period before your periods stopped for 1 year or more?

AGE __ __

**IF UNKNOWN**, CODE AS 99.

J4.	Did your menstrual periods stop because of...

Natural menopause, which means that

the periods stopped by themselves	1

Hysterectomy	2

Removal of both ovaries	3

Radiation or chemotherapy	4

Others(specify)___________________	8

Don’t know	9

**SECTION K. RADIATION EXPOSURE**

*Now I have some questions about x-ray examinations and radiation treatments. First I will ask you about x-ray examinations in the chest area. Please do not include mammograms*.

| K1. Up until one year before your diagnosis of cancer (or one year before interview), did you ever have any of the following types of x-ray examinations? |  | **IF YES:**  K2. How old were you when you first had this type of x-ray examination? | K3. Up until one year before your diagnosis of cancer, how many times did you have this type of x-ray examination? |
| --- | --- | --- | --- |
| X-ray examinations for tuberculosis (TB) | YES 1  NO 2  Don’t know 9 9 | AGE __ __ | __ __ |
| X-ray examinations for scoliosis | YES 1  NO 2  Don’t know 9 9 | AGE __ __ | __ __ |
| Other x-ray examinations of the chest area **(SPECIFY)**  ____________________ | YES 1  NO 2  Don’t know 9 | AGE __ __ | __ __ |

Now I have some questions about radiation treatments that included the chest area. Please do not include radiation treatments you may have received for the treatment of breast cancer.

| K4. Up until one year before your diagnosis of cancer (or one year before interview)), had you ever been treated with radiation for any of the following conditions? |  | **IF YES:**  K5. How old were you when you were first treated with radiation for this condition? | K6. Up until one year before your diagnosis of cancer, how many times had you been treated with radiation for this condition? |
| --- | --- | --- | --- |
| Tuberculosis | YES 1  NO 2  Don’t know 9 9 | AGE __ __ | __ __ __ |
| Cancer | YES 1  NO 2  Don’t know 9 9 | AGE __ __ | __ __ __ |
| Acne | YES 1  NO 2  Don’t know 9 9 | AGE __ __ | __ __ __ |
| Mastitis | YES 1  NO 2  Don’t know 9 9 | AGE __ __ | __ __ __ |
| Enlarged thymus gland | YES 1  NO 2  Don’t know 9 9 | AGE __ __ | __ __ __ |
| Hemangioma | YES 1  NO 2  Don’t know 9 9 | AGE __ __ | __ __ __ |
| Other conditions in the chest area **(SPECIFY)**  _____________________ | YES 1  NO 2  Don’t know 9 9 | AGE __ __ | __ __ __ |

*The next questions are about radiation treatments that included the lower abdomen or pelvis.*

| K7. Up until one year before your diagnosis of cancer (or one year before this interview), had you ever been treated with radiation for any of the following conditions? |  | **IF YES:**  K8. How old were you when you were first treated with radiation for this condition? How old were you when you were first treated with radiation for this condition? | K9. Up until one year before your diagnosis of cancer, how many times had you been treated with radiation for this condition? Up until one year before your diagnosis of cancer, how many times had you been treated with radiation for this condition? |
| --- | --- | --- | --- |
| Cancer | YES 1  NO 2  Don’t know 9 9 | AGE __ __ | __ __ __ |
| Bleeding from the uterus or womb | YES 1  NO 2  Don’t know 9 9 | AGE __ __ | __ __ __ |
| Growth on the uterus or womb | YES 1  NO 2  Don’t know 9 9 | AGE __ __ | __ __ __ |
| Other conditions in the lower abdomen or pelvis **(SPECIFY)**  _____________________ | YES 1  NO 2  Don’t know 9 9 | AGE __ __ | __ __ __ |

**SECTION L. PHYSICAL ACTIVITY**

*The next questions I am going to ask you are about the time you spent being physically active* ***in the past year (or the year before diagnosis of breast cancer for patients)****. Think about the activities you do as part of your house and yard work, to get from place to place, at work, and in your spare time for recreation, exercise or sport.*

*HOUSEWORK-RELATED PHYSICAL ACTIVITY*

*The next questions ask about the physical activities you have done in the past year in and around your home, as part of housework, which also includes gardening, yard work, general maintenance work, and caring your family.*

| L1. Housework schedule | L1a. Did you usually do housework? | Yes 1  No 2  Don’t know 9 |
| --- | --- | --- |
|  | L1b. If you did, what was the average number of days per week that you did housework? | __  days per week |
|  | L1c. During those days, what was the average number of hours per day that you did housework? | __ __  hours per day |
| L2. Housework activities | L2a. How many hours per day did you spend in **light housework**? E.g. cooking, slow walking, light cleaning (such as sweeping and dusting), and all sitting activities. | __ __  hours per day |
|  | L2b. How many hours per day did you spend in **moderate housework**, which may make you breathe somewhat harder than normal. E.g. child care, heavy cleaning (washing clothes, mopping, scrubbing, scraping), carrying loads, and gardening (planting or weeding). | __ __  hours per day |
|  | L2c. How many hours per day did you spend in **vigorous housework**, which may make you breathe much harder than normal or make you sweat. E.g. pounding yam, chopping woods, and farming. | __ __  hours per day |
| L3. Housework related walking | In a typical day of housework, how many minutes did you walk for housework-related purposes (such as from home to market, errands, etc)? | __ __ __  minutes per day |

*OCCUPATIONAL PHYSICAL ACTIVITY*

The first questions are about your work. This includes paid jobs (or unpaid work that you did outside your home) for >1 month in the past year. If you had more than one jobs, let’s go through them one by one:

|  | | 1ST JOB | 2ND JOB | 3RD JOB |
| --- | --- | --- | --- | --- |
| L4. Job title | |  |  |  |
| L5. Job schedule | L2a. In rough terms, how many months out of the 12 months did you work in this job? | __ __  months per year | __ __  months per year | __ __  months per year |
|  | L2b. During those months, what was the average number of days per week that you worked in this job? | __  days per week | __  days per week | __  days per week |
|  | L2c. During those days, what was the average number of hours per day that you worked in this job? | __ __  hours per day | __ __  hours per day | __ __  hours per day |
| L6. Job activities | L3a. For this job, how many hours per day did you spend in **light activities**? E.g. standing, slow walking, and all sitting activities. | __ __  hours per day | __ __  hours per day | __ __  hours per day |
|  | L3b. For this job, how many hours per day did you spend in **moderate activities**, which may make you breathe somewhat harder than normal. E.g. carrying loads, continuous walking as part of your work (Do not count any walking you did to travel to or from work), painting/plastering, and plumbing/welding. | __ __  hours per day | __ __  hours per day | __ __  hours per day |
|  | L3c. For this job, how many hours per day did you spend in **vigorous activities**, which may make you breathe much harder than normal or make you sweat. E.g. heavy construction, digging ditches, farming, chopping (ax), and sawing. | __ __  hours per day | __ __  hours per day | __ __  hours per day |
| L7. Walk to work | In a typical working day, how many minutes did you walk to work? | __ __ __  minutes per day | __ __ __  minutes per day | __ __ __  minutes per day |

*LEISURE-TIME PHYSICAL ACTIVITY*

Please recall all activities you did at least 6 times over the past year for leisure purposes. Do not include activities related to your job or housework.

L8. For each leisure-time activity: please write down the number of month per year, the number of session per month, and the mean duration of a session of each activity performed over the past year.

| ACTIVITY | MONTHS  PER YEAR | SESSIONS  PER MONTH | MINUTES  PER SESSION |
| --- | --- | --- | --- |
| Leisure walking | __ __ | __ __ | __ __ __ |
| Jogging | __ __ | __ __ | __ __ __ |
| Light dancing | __ __ | __ __ | __ __ __ |
| Vigorous dancing | __ __ | __ __ | __ __ __ |
| Sports (e.g. football, handball, etc.) | __ __ | __ __ | __ __ __ |
| Aerobic exercise/class | __ __ | __ __ | __ __ __ |
| Other(s)  SPECIFY _________________ | __ __ | __ __ | __ __ __ |
|  |  |  |  |
|  |  |  |  |
|  |  |  |  |

**SECTION M. MISCELLANEOUS**

M1.	In the past 5 years, have you ever taken continuously the following vitamins at least three times a week for two months? (If there were changes in the past year, please refer to the use before the changes.)

| Name | Multi- vitamins | Vitamin B | Vitamin C | Vitamin D | Vitamin E | Others(specify)  ____________ |
| --- | --- | --- | --- | --- | --- | --- |
| 1. Ever took? | YES 1  NO 2 | YES 1  NO 2 | YES 1  NO 2 | YES 1  NO 2 | YES 1  NO 2 | YES 1  NO 2 |
| 2. How many months in total did you take the vitamins? | \|__\|__\|  month(s) | \|__\|__\|  month(s) | \|__\|__\|  month(s) | \|__\|__\|  month(s) | \|__\|__\|  month(s) | \|__\|__\|  month(s) |
| 3. When you took vitamins, how many times per week did you take them? | \|__\|__\|  times | \|__\|__\|  times | \|__\|__\|  times | \|__\|__\|  times | \|__\|__\|  times | \|__\|__\|  times |

M2.	When you were sick, did you often take or use vitamins?

YES	1

NO	2

Don’t know	9

M3.	Are you a twin?

YES	1

NO	2	**GO TO M5.**

Don’t know	9	**GO TO M5.**

**IF YES:**

M4.	Non-identical twins are no more alike physically than ordinary brothers and sisters. Genetically identical twins, on the other hand, have a strong resemblance to each other in height, coloring, features of the face, etc. They look so much alike that people often mistake one for the other, especially during their childhood.

Do you think you and your twin are identical?

YES	1

NO	2

Don’t know	9

M5. Have you ever taken any traditional medication (before breast cancer diagnosis, if any)?

YES	1

NO	2

Don’t know	9

M6. For what purpose did you take traditional medication?

Backache	1

Fever	2

Body weakness	3

Low blood level (anemia)	4

Pregnancy	5

Prevention of diseases	6

Don’t know	9

Treatment of other diseases	8

(SPECIFY)

_________________________

M7. On average, how often do you take traditional medication?

Everyday	1

2-6 times a week	2

Once a week	3

< Once a week, but ≥ once a month	4

< Once a month	5

Don’t know	9

**SECTION N. Family Cancer History**

# Cancer History in Mother and Grandmothers

|  | Mother | Mother’s Mother | Father’s Mother |
| --- | --- | --- | --- |
| **N1.** Is she still living?  **IF YES:**  **N2a.** How old is she?  **IF NO:**  **N2b.** How old was she when she died? | YES 1  NO 2  Don’t know 9  AGE __ __  AGE __ __ | YES 1  NO 2  Don’t know 9  AGE __ __  AGE __ __ | YES 1  NO 2  Don’t know 9  AGE __ __  AGE __ __ |
| **N3.** Did she ever have cancer?  **IF YES:**  **N4.** What type(s) of cancer did she have? And how old was she when this cancer was first diagnosed?  Breast 1 __ __  Ovary 2 __ __  Cervical 3 __ __  Uterine/Endometrial 4 __ __  Colon 5 __ __  Lung 6 __ __  Don’t know 9 __ __  Other 88 __ __ **(SPECIFY)**  __________________________  **(*Code 90 if she had cancer and site is unknown, but not breast.)***  **IF Breast cancer:**  **N5.** Did she have it in one or both breasts? | YES 1  NO 2  Don’t know 9  TYPE AGE  __ __  __ __  __ __  __ __  __ __  __ __  __ __  __ __  One 1  Both 2  Don’t know 9 | YES 1  NO 2  Don’t know 9  TYPE AGE  __ __  __ __  __ __  __ __  __ __  __ __  __ __  __ __  One 1  Both 2  Don’t know 9 | YES 1  NO 2  Don’t know 9  TYPE AGE  __ __  __ __  __ __  __ __  __ __  __ __  __ __  __ __  One 1  Both 2  Don’t know 9 |

*Cancer History in Sisters*

|  | Sister #1 | Sister #2 | Sister #3 | Sister #4 | Sister #5 | Sister #6 |
| --- | --- | --- | --- | --- | --- | --- |
| Sister initials |  |  |  |  |  |  |
| **N6.** Is this sister a full or half sister? | Full 1  Maternal half 2  Paternal half 3  Don’t know 9 | Full 1  Maternal half 2  Paternal half 3  Don’t know 9 | Full 1  Maternal half 2  Paternal half 3  Don’t know 9 | Full 1  Maternal half 2  Paternal half 3  Don’t know 9 | Full 1  Maternal half 2  Paternal half 3  Don’t know 9 | Full 1  Maternal half 2  Paternal half 3  Don’t know 9 |
| **N7.** Is this sister still living?  **IF YES:**  **N8a.** How old is she?  **IF NO:**  **N8b.** How old was she when she died? | YES 1  NO 2  Don’t know 9  AGE __ __  AGE __ __ | YES 1  NO 2  Don’t know 9  AGE __ __  AGE __ __ | YES 1  NO 2  Don’t know 9  AGE __ __  AGE __ __ | YES 1  NO 2  Don’t know 9  AGE __ __  AGE __ __ | YES 1  NO 2  Don’t know 9  AGE __ __  AGE __ __ | YES 1  NO 2  Don’t know 9  AGE __ __  AGE __ __ |
| **N9.** Did she ever have cancer?  **IF YES:**  **N10.** What type(s) of cancer did she have? And how old was she when this cancer was first diagnosed?  Breast 1  Ovary 2  Cervical 3  Uterine/Endometrial 4  Colon 5  Lung 6  Don’t know 9  Other 88  **(SPECIFY)**  _________________________  **(*Code 90 if she had cancer and site is unknown, but not breast.)***  **IF Breast cancer:**  **N11.** Did she have it in one or both breasts? | YES 1  NO 2  Don’t know 9  TYPE AGE  __ __  __ __  __ __  __ __  __ __  __ __  __ __  __ __  One 1  Both 2  Don’t know 9 | YES 1  NO 2  Don’t know 9  TYPE AGE  __ __  __ __  __ __  __ __  __ __  __ __  __ __  __ __  One 1  Both 2  Don’t know 9 | YES 1  NO 2  Don’t know 9  TYPE AGE  __ __  __ __  __ __  __ __  __ __  __ __  __ __  __ __  One 1  Both 2  Don’t know 9 | YES 1  NO 2  Don’t know 9  TYPE AGE  __ __  __ __  __ __  __ __  __ __  __ __  __ __  __ __  One 1  Both 2  Don’t know 9 | YES 1  NO 2  Don’t know 9  TYPE AGE  __ __  __ __  __ __  __ __  __ __  __ __  __ __  __ __  One 1  Both 2  Don’t know 9 | YES 1  NO 2  Don’t know 9  TYPE AGE  __ __  __ __  __ __  __ __  __ __  __ __  __ __  __ __  One 1  Both 2  Don’t know 9 |

*Cancer History in Mother’s Sister*

|  | Mother’s Sister #1 | Mother’s Sister #2 | Mother’s Sister #3 | Mother’s Sister #4 | Mother’s Sister #5 | Mother’s Sister #6 |
| --- | --- | --- | --- | --- | --- | --- |
| Mother’s sister initials |  |  |  |  |  |  |
| **N12.** Is this Mother’s sister a full or half sister? | Full 1  Maternal half 2  Paternal half 3  Don’t know 9 | Full 1  Maternal half 2  Paternal half 3  Don’t know 9 | Full 1  Maternal half 2  Paternal half 3  Don’t know 9 | Full 1  Maternal half 2  Paternal half 3  Don’t know 9 | Full 1  Maternal half 2  Paternal half 3  Don’t know 9 | Full 1  Maternal half 2  Paternal half 3  Don’t know 9 |
| **N13**. Is your Mother’s sister still living?  **IF YES:**  **N14a.** How old is she?  **IF NO:**  **N14b.** How old was she when she died? | YES 1  NO 2  Don’t know 9  AGE __ __  AGE __ __ | YES 1  NO 2  Don’t know 9  AGE __ __  AGE __ __ | YES 1  NO 2  Don’t know 9  AGE __ __  AGE __ __ | YES 1  NO 2  Don’t know 9  AGE __ __  AGE __ __ | YES 1  NO 2  Don’t know 9  AGE __ __  AGE __ __ | YES 1  NO 2  Don’t know 9  AGE __ __  AGE __ __ |
| **N15.** Did she ever have cancer?  **IF YES:**  **N16.** What type(s) of cancer did she have? And how old was she when this cancer was first diagnosed?  Breast 1  Ovary 2  Cervical 3  Uterine/Endometrial 4  Colon 5  Lung 6  Don’t know 9  Other 88  **(SPECIFY)**  _________________________  **(*Code 90 if she had cancer and site is unknown, but not breast.)***  **IF Breast cancer:**  **N17.** Did she have it in one or both breasts? | YES 1  NO 2  Don’t know 9  TYPE AGE  __ __  __ __  __ __  __ __  __ __  __ __  __ __  __ __  One 1  Both 2  Don’t know 9 | YES 1  NO 2  Don’t know 9  TYPE AGE  __ __  __ __  __ __  __ __  __ __  __ __  __ __  __ __  One 1  Both 2  Don’t know 9 | YES 1  NO 2  Don’t know 9  TYPE AGE  __ __  __ __  __ __  __ __  __ __  __ __  __ __  __ __  One 1  Both 2  Don’t know 9 | YES 1  NO 2  Don’t know 9  TYPE AGE  __ __  __ __  __ __  __ __  __ __  __ __  __ __  __ __  One 1  Both 2  Don’t know 9 | YES 1  NO 2  Don’t know 9  TYPE AGE  __ __  __ __  __ __  __ __  __ __  __ __  __ __  __ __  One 1  Both 2  Don’t know 9 | YES 1  NO 2  Don’t know 9  TYPE AGE  __ __  __ __  __ __  __ __  __ __  __ __  __ __  __ __  One 1  Both 2  Don’t know 9 |

*Cancer History in Father’s Sister*

|  | Father’s Sister #1 | Father’s Sister #2 | Father’s Sister #3 | Father’s Sister #4 | Father’s Sister #5 | Father’s Sister #6 |
| --- | --- | --- | --- | --- | --- | --- |
| Father’s sister initials |  |  |  |  |  |  |
| **N18.** Is this Father’s sister a full or half sister? | Full 1  Maternal half 2  Paternal half 3  Don’t know 9 | Full 1  Maternal half 2  Paternal half 3  Don’t know 9 | Full 1  Maternal half 2  Paternal half 3  Don’t know 9 | Full 1  Maternal half 2  Paternal half 3  Don’t know 9 | Full 1  Maternal half 2  Paternal half 3  Don’t know 9 | Full 1  Maternal half 2  Paternal half 3  Don’t know 9 |
| **N19**. Is your Father’s sister still living?  **IF YES:**  **N20a.** How old is she?  **IF NO:**  **N20b.** How old was she when she died? | YES 1  NO 2  Don’t know 9  AGE __ __  AGE __ __ | YES 1  NO 2  Don’t know 9  AGE __ __  AGE __ __ | YES 1  NO 2  Don’t know 9  AGE __ __  AGE __ __ | YES 1  NO 2  Don’t know 9  AGE __ __  AGE __ __ | YES 1  NO 2  Don’t know 9  AGE __ __  AGE __ __ | YES 1  NO 2  Don’t know 9  AGE __ __  AGE __ __ |
| **N21.** Did she ever have cancer?  **IF YES:**  **N22.** What type(s) of cancer did she have? And how old was she when this cancer was first diagnosed?  Breast 1  Ovary 2  Cervical 3  Uterine/Endometrial 4  Colon 5  Lung 6  Don’t know 9  Other 88  **(SPECIFY)**  _________________________  **(*Code 90 if she had cancer and site is unknown, but not breast.)***  **IF Breast cancer:**  **N23.** Did she have it in one or both breasts? | YES 1  NO 2  Don’t know 9  TYPE AGE  __ __  __ __  __ __  __ __  __ __  __ __  __ __  __ __  One 1  Both 2  Don’t know 9 | YES 1  NO 2  Don’t know 9  TYPE AGE  __ __  __ __  __ __  __ __  __ __  __ __  __ __  __ __  One 1  Both 2  Don’t know 9 | YES 1  NO 2  Don’t know 9  TYPE AGE  __ __  __ __  __ __  __ __  __ __  __ __  __ __  __ __  One 1  Both 2  Don’t know 9 | YES 1  NO 2  Don’t know 9  TYPE AGE  __ __  __ __  __ __  __ __  __ __  __ __  __ __  __ __  One 1  Both 2  Don’t know 9 | YES 1  NO 2  Don’t know 9  TYPE AGE  __ __  __ __  __ __  __ __  __ __  __ __  __ __  __ __  One 1  Both 2  Don’t know 9 | YES 1  NO 2  Don’t know 9  TYPE AGE  __ __  __ __  __ __  __ __  __ __  __ __  __ __  __ __  One 1  Both 2  Don’t know 9 |

*Cancer History in Daughters*

|  | Daughter #1 | Daughter #2 | Daughter #3 | Daughter #4 | Daughter #5 | Daughter #6 |
| --- | --- | --- | --- | --- | --- | --- |
| Daughter initials |  |  |  |  |  |  |
| **N24**. Is your daughter still living?  **IF YES:**  **N25a.** How old is she?  **IF NO:**  **N25b.** How old was she when she died? | YES 1  NO 2  Don’t know 9  AGE __ __  AGE __ __ | YES 1  NO 2  Don’t know 9  AGE __ __  AGE __ __ | YES 1  NO 2  Don’t know 9  AGE __ __  AGE __ __ | YES 1  NO 2  Don’t know 9  AGE __ __  AGE __ __ | YES 1  NO 2  Don’t know 9  AGE __ __  AGE __ __ | YES 1  NO 2  Don’t know 9  AGE __ __  AGE __ __ |
| **N26.** Did she ever have cancer?  **IF YES:**  **N27.** What type(s) of cancer did she have? And how old was she when this cancer was first diagnosed?  Breast 1  Ovary 2  Cervical 3  Uterine/Endometrial 4  Colon 5  Lung 6  Don’t know 9  Other 88  **(SPECIFY)**  _________________________  **(*Code 90 if she had cancer and site is unknown, but not breast.)***  **IF Breast cancer:**  **N28.** Did she have it in one or both breasts? | YES 1  NO 2  Don’t know 9  TYPE AGE  __ __  __ __  __ __  __ __  __ __  __ __  __ __  __ __  One 1  Both 2  Don’t know 9 | YES 1  NO 2  Don’t know 9  TYPE AGE  __ __  __ __  __ __  __ __  __ __  __ __  __ __  __ __  One 1  Both 2  Don’t know 9 | YES 1  NO 2  Don’t know 9  TYPE AGE  __ __  __ __  __ __  __ __  __ __  __ __  __ __  __ __  One 1  Both 2  Don’t know 9 | YES 1  NO 2  Don’t know 9  TYPE AGE  __ __  __ __  __ __  __ __  __ __  __ __  __ __  __ __  One 1  Both 2  Don’t know 9 | YES 1  NO 2  Don’t know 9  TYPE AGE  __ __  __ __  __ __  __ __  __ __  __ __  __ __  __ __  One 1  Both 2  Don’t know 9 | YES 1  NO 2  Don’t know 9  TYPE AGE  __ __  __ __  __ __  __ __  __ __  __ __  __ __  __ __  One 1  Both 2  Don’t know 9 |

*Cancer History in Male Relatives*

**N29.** Were any of your male relatives, specifically your grandfathers, your father,

your uncles, your brothers or your sons, ever diagnosed with cancer?

YES	1

NO	2	**GO TO N31**

Don’t know	9	**GO TO N31**

**IF YES:**

**N3**0.

| a. Which of your relatives was diagnosed with cancer? (Code) | b. What type of cancer does/did he have? (Code) | c. How old was he when he was first diagnosed? |
| --- | --- | --- |
|  |  |  |
|  |  |  |
|  |  |  |
|  |  |  |
|  |  |  |
|  |  |  |
|  |  |  |
|  |  |  |

Male Relative Codes

| 51 | Father | 59 | Half brother of mother (maternal side) |
| --- | --- | --- | --- |
| 52 | Maternal grandfather | 60 | Half brother of mother (paternal side) |
| 53 | Paternal grandfather | 61 | Half brother of mother (unknown side) |
| 54 | Full brother | 62 | Full brother of father |
| 55 | Half brother (maternal side) | 63 | Half brother of father (maternal side) |
| 56 | Half brother (paternal side) | 64 | Half brother of father (paternal side) |
| 57 | Half brother (unknown side) | 65 | Half brother of father (unknown side) |
| 58 | Full brother of mother | 66 | Son |

##	Male Cancer Type Codes

| 1 | Breast | 5 | Pancreas |
| --- | --- | --- | --- |
| 2 | Lung | 9 | Don’t know |
| 3 | Colon | 88 | Other  __________ |
| 4 | Prostate |  |  |

**N31.** How many full and half brothers do you have? Please include both living and deceased brothers.

N31a	. # of Full Brothers ___

N31b. # of Maternal Half Brothers ___

N31c. # of Paternal Half Brothers ____

**N32.** How many of these brothers reached age 20 or more?

N32a. # of Full Brothers ___

N32b. # of Maternal Half Brothers ___

N32c. # of Paternal Half Brothers ____

**END:**	Thank you very much for taking the time to complete this interview.

**CONCLUSION**

This concludes the questionnaire. We would like to get a few more pieces of information that will help us locate you in the future. We would appreciate if you would please record the name, address and telephone number of one or more close friends and relatives who do not live with you, but will always know where you are, in case we need to contact you in the future.

**YourSelf**

Name­­­­­­­­­­­­­­­­­­­­­­___________________________________________

Street___________________________________________

________________________________________

Town/City_______________________________________

State___________________________________________

Telephone number(s)	___________________________

__________________________

**Person 1**

Name­­­­­­­­­­­­­­­­­­­­­­___________________________________________

Street___________________________________________

________________________________________

Town/City_______________________________________

State___________________________________________

Telephone number(s) ______________________________

__________________________

What is this person’s relationship to you?

Relationship________________________________

**Person 2**

Name­­­­­­­­­­­­­­­­­­­­­­___________________________________________

Street___________________________________________

________________________________________

Town/City_______________________________________

State___________________________________________

Telephone number(s) ______________________________

__________________________

What is this person’s relationship to you?

Relationship________________________________

**TIME USED TO COMPLETE INTERVIEW:**	__ __	__ __

HRS	MINS

__ __	__ __

AM	PM

**INTERVIEWER ASSESSMENT**

1.	PARTICIPANT’S COOPERATION WAS

VERY GOOD	1

GOOD	2

FAIR	3

POOR	4

2.	THE OVERALL QUALITY OF THIS INTERVIEW IS

HIGH QUALITY	1

GENERALLY RELIABLE	2

QUESTIONABLE	3

UNSATISFACTORY	4

3.	WERE THERE ANY DISTRACTIONS DURING THE INTERVIEW

YES	1

NO	2

**IF YES**:

DESCRIBE	___________________________________________________

___________________________________________________

___________________________________________________
